# Iron Isotopic Composition of Biological Standards Relevant to Medical and Biological Applications

**DOI:** 10.3389/fmed.2021.696367

**Published:** 2021-10-20

**Authors:** Edith Kubik, Frédéric Moynier, Marine Paquet, Julien Siebert

**Affiliations:** ^1^Université de Paris, Institut de Physique du Globe de Paris, CNRS, Paris, France; ^2^Institut Universitaire de France, Paris, France

**Keywords:** iron isotopes, biological standard reference material, isotope fractionation, iron homeostasis, isotope metallomics

## Abstract

Iron isotopes are fractionated by multiple biological processes, which offers a novel opportunity to study iron homeostasis. The determination of Fe isotope composition in biological samples necessitates certified biological reference materials with known Fe isotopic signature in order to properly assess external reproducibility and data quality between laboratories. We report the most comprehensive study on the Fe isotopic composition for widely available international biological reference materials. They consist of different terrestrial and marine animal organs (bovine, porcine, tuna, and mussel) as well as apple leaves and human hair (ERC-CE464, NIST1515, ERM-DB001, ERM-BB186, ERM-BB184, ERM-CE196, BCR668, ERM-BB185, ERM-BB124). Previously measured Fe isotopic compositions were available for only two of these reference materials (ERC-CE464 tuna fish and ERM-BB186 pig kidney) and these literature data are in excellent agreement with our data. The Fe isotopic ratios are reported as the permil deviation of the ^56^Fe/^54^Fe ratio from the IRMM-014 standard. All reference materials present δ^56^Fe ranging from −2.27 to −0.35%0. Combined with existing data, our results suggest that animal models could provide useful analogues of the human body regarding the metabolic pathways affecting Fe isotopes, with many potential applications to medicine.

## Introduction

An isotope fractionation is defined by a difference in the relative abundances of the isotopes of an element between two reservoirs. Its existence stems from bond energy between atoms which is proportional to the vibrational frequency and therefore increases with the isotope mass. This means that the energy of a system is minimal when the heavier isotopes are stored in the lowest and more stable energy levels (1), corresponding to environments where the bonds are the stiffest [e.g., (2, 3)]. Generally, the strength of a bond increases as the size of an ion and the number of atoms involved in the bond become minimal, and as its charge increases. In this framework, heavy isotopes are favoured over light isotopes in bonds involving high oxidation states and small coordination numbers.

Variations of natural stable isotopes have been used to track a wide range of natural processes, including both inorganic and organic processes (4). In particular, the isotopic behaviour of elements such as Ca, Zn, Cu and Fe during biological processes has recently shown promising results as proxies for transport mechanisms or for diagnosis of diseases affecting the homeostasis of these elements [e.g., (4–27)]. Major recent advances include the potential early diagnosis of osteoporosis from the Ca isotope composition of blood and urine (11), and the potential detection of Alzheimer's disease markers traced by the Cu isotope composition of the serum (21, 28). Moreover, significant differences between healthy patients and cancer patients blood samples have been detected for S and Cu isotopes and for several types of cancers (7, 26), suggesting that the study of metal isotope fractionation could be used for diagnosis as well as investigation of metabolic processes associated to cancer (29, 30). Among these elements, Fe plays a central role as it corresponds to the most abundant metal in the human body and has a turnover time of several years (31, 32).

Iron forms the sites of oxygen binding in Fe(II)-bearing haemoglobin metalloproteins, which transport oxygen and carbon dioxide in blood to and from organs and in the muscles of most vertebrates, making it a key element in evolved animal life on Earth. Liver, spleen and kidney also contain significant amounts of Fe, mainly stored as Fe(III) ferritin, and iron has a major importance in numerous biological processes (e.g., cellular respiration and DNA synthesis).

Iron has four stable isotopes: ^54^Fe, ^56^Fe, ^57^Fe and ^58^Fe. Iron isotope compositions are usually represented as the permil deviation of the ^56^Fe/^54^Fe and ^57^Fe/^54^Fe ratios from a standard (IRMM-014) as:


(1) $$\delta^{56}{\text{Fe}}_{\text{sample}}=\left(\frac{{\left(\frac{{}^{56}\text{Fe}}{{}^{54}\text{Fe}}\right)}_{\text{sample}}}{{\left(\frac{{}^{56}\text{Fe}}{{}^{54}\text{Fe}}\right)}_{\text{IRMM}-014}}-1\right)1000$$

(2) $$\delta^{57}{\text{Fe}}_{\text{sample}}=\left(\frac{{\left(\frac{{}^{57}\text{Fe}}{{}^{54}\text{Fe}}\right)}_{\text{sample}}}{{\left(\frac{{}^{57}\text{Fe}}{{}^{54}\text{Fe}}\right)}_{\text{IRMM}-014}}-1\right)1000$$

**Figure 1:**
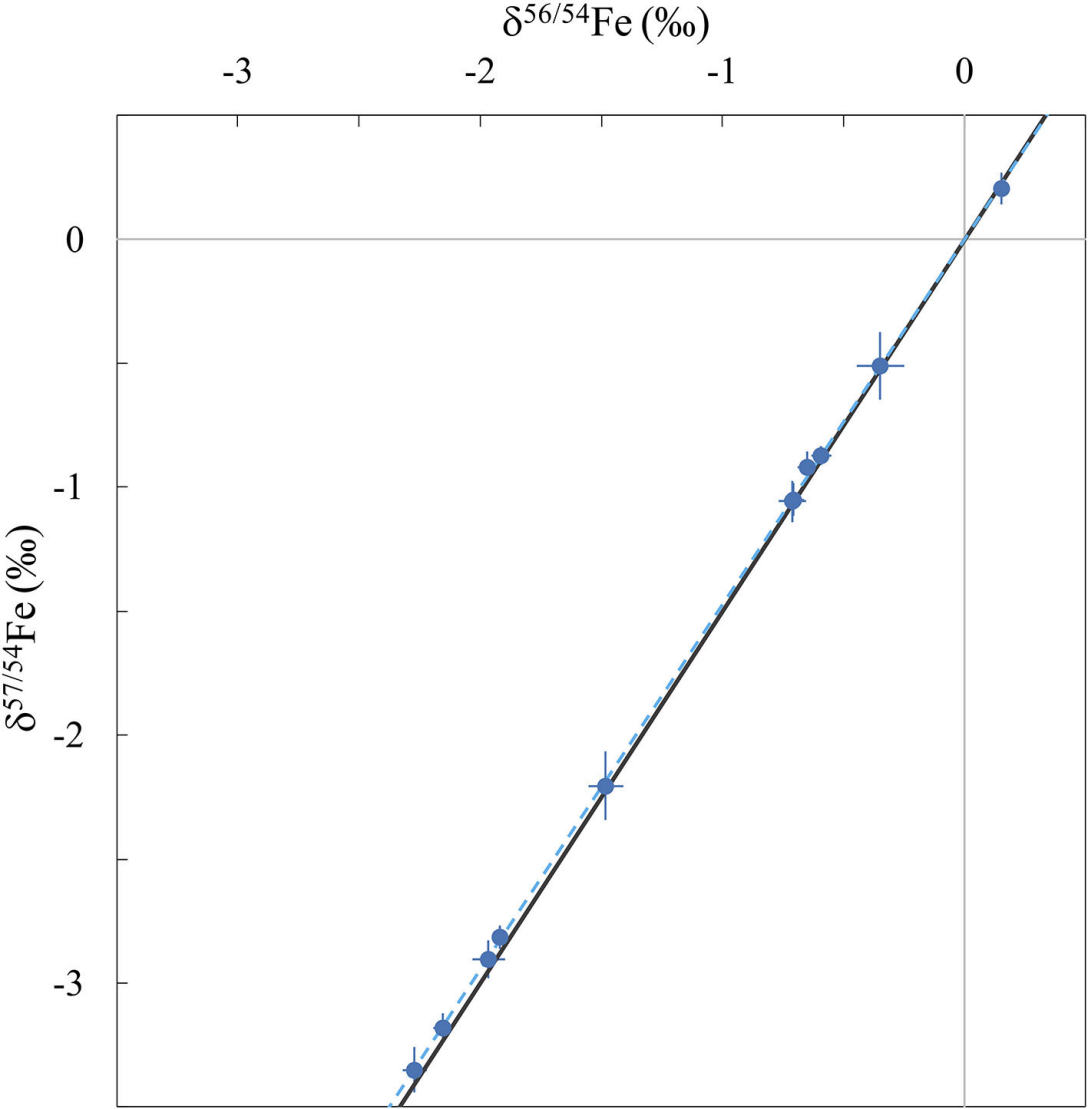
Three-isotope diagram showing δ^57^Fe vs. δ^56^Fe for all samples measured in this study (blue symbols) with 2 s.e. error bars. The blue dotted line corresponds to the regression fit calculated from the dataset and the black line is the theoretical mass-dependent fractionation line.

As the stable isotopic fractionation is dependent of the mass difference between the isotopes (33), typically δ^57^Fe ≈ 1.5 × δ^56^Fe which is validated here (Figure 1). Therefore, all the data will be discussed in terms of δ^56^Fe. δ^56^Fe values have previously been reported for a large variety of biological samples and have given insight into the mechanisms of Fe transports in plants (20, 34, 35), and in animals and humans (6, 36–38). The major source of Fe isotopic fractionation is oxidation–reduction (redox) reactions which have the property of changing the oxidation state of Fe (i.e., Fe^2+^ ↔ Fe^3+^) with the reduced phases enriched in the lighter isotopes of Fe compared to more oxidised phases (39). However, Fe isotopic fractionation is not limited to redox reactions, and more generally Fe isotopes fractionate between all phases in which Fe has different bonding environments (20, 40). This Fe behaviour has led to many isotopic studies, including the application of stable Fe isotopes to detect hereditary hematochromatosis (14).

Therefore, iron isotopes can be used as tracers of numerous metabolic pathways including Fe absorption by the intestine, the storage of Fe in the liver and the synthesis of haemoglobin and myoglobin in erythrocytes. In particular, it has been used to trace the Fe intestinal absorption efficiency (37, 38, 41, 42), the menstrual status (43), genetic polymorphisms (44), and several diseases affecting Fe metabolism, namely malaria, thalassemia, hemochromatosis, and chronic kidney illness (14, 44–49).

Measurements of stable isotopic composition by multi-collection inductively-coupled-plasma mass-spectrometry (MC-ICPMS) are preceded by the digestion of the sample and purification of the element of interest by ion-exchange chromatography. Quality control of stable isotopic measurements necessitates well-characterised and widely available reference materials, with a matrix similar to the matrix of the sample of interest. Although research fields such as geochemistry benefit from numerous extensively characterised international reference rock materials, metal isotope compositions of certified reference materials of biological origin are very scarce [e.g., (50–52)]. In this context, we have determined the Fe isotope composition in nine widely available international biological standards. For seven of them, there is no existing published value for Fe isotope composition. Moreover, due to occasional or definitive unavailability of the international standards such as IRMM-014 which is used as reference, well-characterised biological reference materials are essential to compare different datasets. This data could therefore be used in future biological and/or medical studies to cross calibrate values between laboratories and as quality control.

## Methods

The samples consist of nine international biological reference standards: NIST1515 (apple leaves), ERC-CE464 (tuna fish), BCR668 (mussel tissue), ERM-BB184 (bovine muscle), ERM-CE196 (bovine blood), ERM-BB185 (bovine liver), ERM-BB124 (pork muscle), ERM-BB186 (pig kidney), ERM-DB001 (human hair). The United States Geological Survey (USGS) BHVO-2 (basalt, Kilauea, Hawaii, USA) was also processed and measured to further assess external reproducibility as this sample has been analysed multiple times by various groups.

Prior to measurement, sample digestion and purification were performed at the Institut de Physique du Globe de Paris in a class-100 clean room with class-10 laminar flow hoods. The acids used in this work were distillated from BASF Selectipur^®^ AR grade acids (69% HNO_3_; 37% HCl). All dilutions used ultra-pure (18.2 MΩ cm purity) Milli-Q water. Disposable supplies (pipette tips, columns and test tubes) and Savillex Teflon PFA beakers used for the chemical protocol were washed in a 30% concentrated HNO_3_ bath for 3–5 days. Beakers were further cleaned with 1 to 2 cycles of high-temperature refluxes using concentrated HCl and concentrated HF/HNO_3_ at 130°C for a minimum of 24 h per cycle.

Between 150 and 500 mg of each biological standard were weighed in pre-cleaned beakers and digested into 3 mL of concentrated HNO_3_ with ~1 mL of H_2_O_2_. A full replicate sample was prepared for NIST1515 (apple leaves) and two procedural blanks were applied the same protocol as the samples. The samples were vented until complete reaction of the acid mixture with organic matter. It should be noted that aliquots from these dissolutions were used in another study on the K isotopic composition of biological materials (53). They were then further digested in closed beakers at 100°C, and subsequently dried down after complete digestion and 2 mL of 6 N HCl were added to prepare them for purification. The Fe fraction of the samples was separated from the matrix using the method described in Dauphas and Rouxel (54, 55). Anion exchange chromatography was performed in Biorad^®^ 0.8 × 4 cm columns filled with 1 mL AG1-X8 resin (200–400 mesh). The resin was cleaned with 20 mL Milli-Q, 5 mL 1 N HNO_3_, and 10 mL 0.4 N HCl and conditioned with 5 mL 6 N HCl. After sample loading, the matrix was eluted with 12 mL 6 N HCl. The Fe fractions were collected in 13.5 mL 0.4 N HCl and dried down. They were redissolved in 0.5 N HNO_3_ for measurement, by addition of concentrated nitric acid until complete digestion of the Fe cut prior to the addition of adequate volumes of Milli-Q water for dilution. This protocol yielded procedural blanks inferior to 10 ng (*n* = 2), representing <0.2% of the purified samples (always more than 1 μg of Fe).

Iron isotopic compositions were determined using a Thermo-Scientific Neptune Plus MC-ICPMS at the Institut de Physique du Globe de Paris (Université de Paris). The cups were configured as to collect the signal of ^54^Fe, ^56^Fe, ^57^Fe, ^58^Fe, ^60^Ni and ^53^Cr into Faraday collectors connected to 10^11^ Ω amplifiers. Chromium measurements were used to correct for any ^54^Cr interference on ^54^Fe, even though the amount of Cr after chromatographic separation lead to insignificant correction. All operating and measurement conditions are summarised in Table 1. The instrument was used in high resolution mode, and the samples were introduced with an ESI Apex-IR desolvator with a 100 μL min^−1^ PFA nebuliser.

**Table 1:** Instrumental operating conditions and measurement parameters for the Neptune Plus MC-ICPMS.

**Instrument operating conditions**	
**RF power**	**1,300 W**
**Plasma cool gas flow rate**	**16 L min^−1^**
**Interface cones**	**Jet cone (sampler), Ni skimmer H-type (skimmer)**
**Source slit widtd**	**0.25 mm**
**Acceleration voltage**	**10 kV**
**Instrument resolution**	**High**
**Mass analyser pressure**	**ca. 8 × 10^−9^ mbar**
**Detector**	**9 Faraday detectors**
**Sample introduction system**	**ESI Apex IR**
**Sample uptake rate**	**100 μL min^−1^**
**Measurement parameters**	
**Solution concentration**	**1 μg mL^−1^**
**Typical sensitivity**	**~12 V ppm^−1^ ^56^Fe**
**Sample measurement time**	**60 s**
**Cycles**	**25**
**Washout time**	**100 s**

A 1 ppm solution of IRMM-014 was used for external standardisation of the sample measurements. This solution was measured between each sample (sample–standard bracketing) and the average from IRMM-014 ratios measured before and after each sample was used to calculate the δ^56^Fe and δ^57^Fe reported in this study.

## Results and Discussion

The Fe isotope composition of the nine international biological standards and USGS rock standard BHVO-2 are reported in Table 2. Figure 1 represents the δ^57^Fe as a function of δ^56^Fe values of all samples. This three-isotope plot allows to assess the mass fractionation relationship and calculate the linear regression fit and associated R^2^ value. The regression coefficient shows excellent agreement with theoretical values for mass-dependent isotopic fractionation (δ^57^Fe = 1.47 × δ^56^Fe, blue dotted line on Figure 1), demonstrating that the Fe isotopic compositions measured in the samples were induced by mass-dependent fractionation processes (R^2^ = 0.9998).

**Table 2:** Iron isotopic compositions of nine biological standards.

**Sample**	**Type**	**n**	**δ^56^Fe (%0)**	**2 s.e**.	**2 s.d**.	**δ^57^Fe (%0)**	**2 s.e**.	**2 s.d**.
NIST1515	Apple leaves	6	−0.71	0.06	0.14	−1.06	0.08	0.20
NIST1515	Apple leaves	6	−0.71	0.04	0.10	−1.05	0.07	0.16
ERC-CE464	Tuna fish	6	−0.65	0.04	0.10	−0.92	0.06	0.15
BCR668	Mussel tissue	6	−0.59	0.04	0.10	−0.87	0.04	0.10
ERM-BB184	Bovine muscle	6	−1.97	0.07	0.17	−2.91	0.08	0.19
ERM-CE196	Bovine blood	6	−2.27	0.05	0.12	−3.35	0.09	0.22
ERM-BB185	Bovine liver	6	−1.48	0.07	0.17	−2.20	0.14	0.34
ERM-BB124	Pork muscle	6	−1.92	0.02	0.04	−2.82	0.05	0.11
ERM-BB186	Pig kidney	6	−2.16	0.04	0.09	−3.18	0.06	0.14
ERM-DB001	Human hair	6	−0.35	0.10	0.24	−0.51	0.14	0.33
BHVO-2	Basalt	5	0.15	0.03	0.07	0.20	0.06	0.16

*Errors are given as standard deviations (s.d.) and standard errors (s.e.) calculated from the number of single measurements (n) with s.e=s.d./√n*.

**Figure 2:**
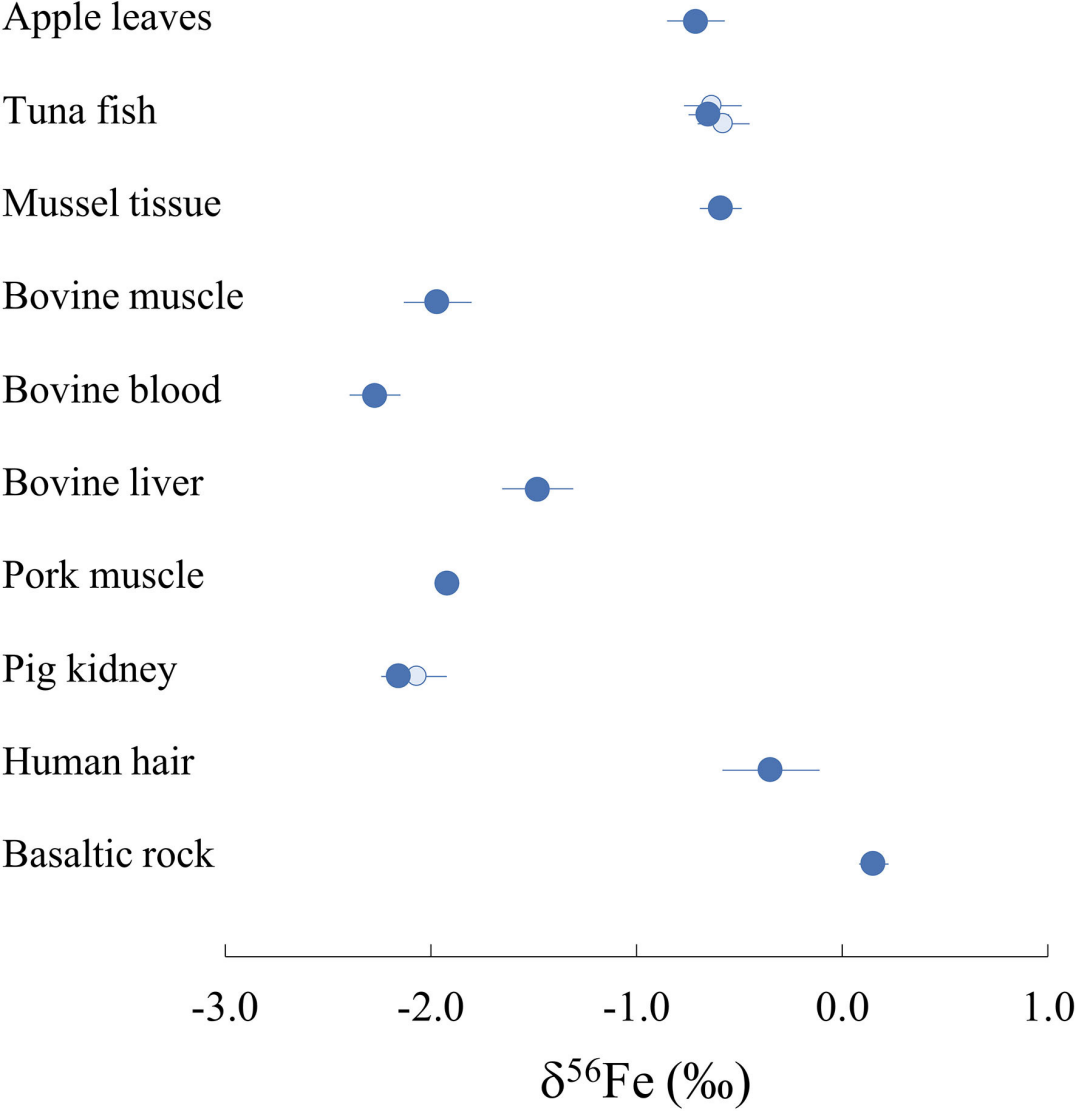
Iron isotope composition δ^56^Fe of all international biological reference materials measured in this study and USGS BHVO-2 rock standard (dark blue). Existing published data are also represented with light blue circles [(51) for pig kidney; (52) for tuna fish] for two of the reference materials measured. Represented error bars correspond to 2 s.d.

The δ^56^Fe values of all samples measured in this study range from −2.27 to −0.35%0 and are displayed on Figure 2. The two full replicates measured for NIST1515, which underwent identical procedures in separate beakers from the digestion process, present identical values (−0.71 ± 0.06%0 and −0.71 ± 0.04%0). Two samples (ERC-CE464, tuna fish and ERM-BB186, pig kidney) had previously been analysed (51, 52) and our data are in very good agreement with previously reported values (Figure 2). The isotopic composition of BHVO-2 is within error of previously published values [0.17 ± 0.03%0 for δ^57^Fe and 0.10 ± 0.04%0 for δ^56^Fe, e.g., (56)]. The routine precision for δ^56^Fe is below 100 ppm which is adequate in order to distinguish natural isotope variations in samples and in line with the state-of-the-art precision found in previous studies studies [e.g., (57, 58)].

All reference materials of biological origin measured in this study present lighter Fe isotope compositions than standard IRMM-014, resulting in negative δ^56^Fe values. The dataset can be divided into two groups with human hair (ERM-DB001), apple leaves (NIST1515) and organs of marine animal origin (tuna fish ERC-CE464 and mussel tissue BCR668) presenting δ^56^Fe values ranging from −0.35 to −0.71%0 (Figure 2). The five reference materials sampled from bovine and porcine organs present more fractioned Fe isotope compositions compared to the IRMM-014 standard, with δ^56^Fe values from −1.48 to −2.27%0. Pork (ERM-BB124) and bovine (ERM-BB184) muscles present very close δ^56^Fe values of −1.92 ± 0.02%0 and −1.97 ± 0.07%0 respectively suggesting that iron fractionation processes in both organisms are similar. On the other hand, bovine blood (ERM-CE196) and liver (ERM-BB185) yield significantly different δ^56^Fe values of −2.27 ± 0.12%0 and −1.48 ± 0.17%0, respectively, suggesting the existence of one or several processes fractionating Fe isotopes between the three biological reservoirs.

**Figure 3:**
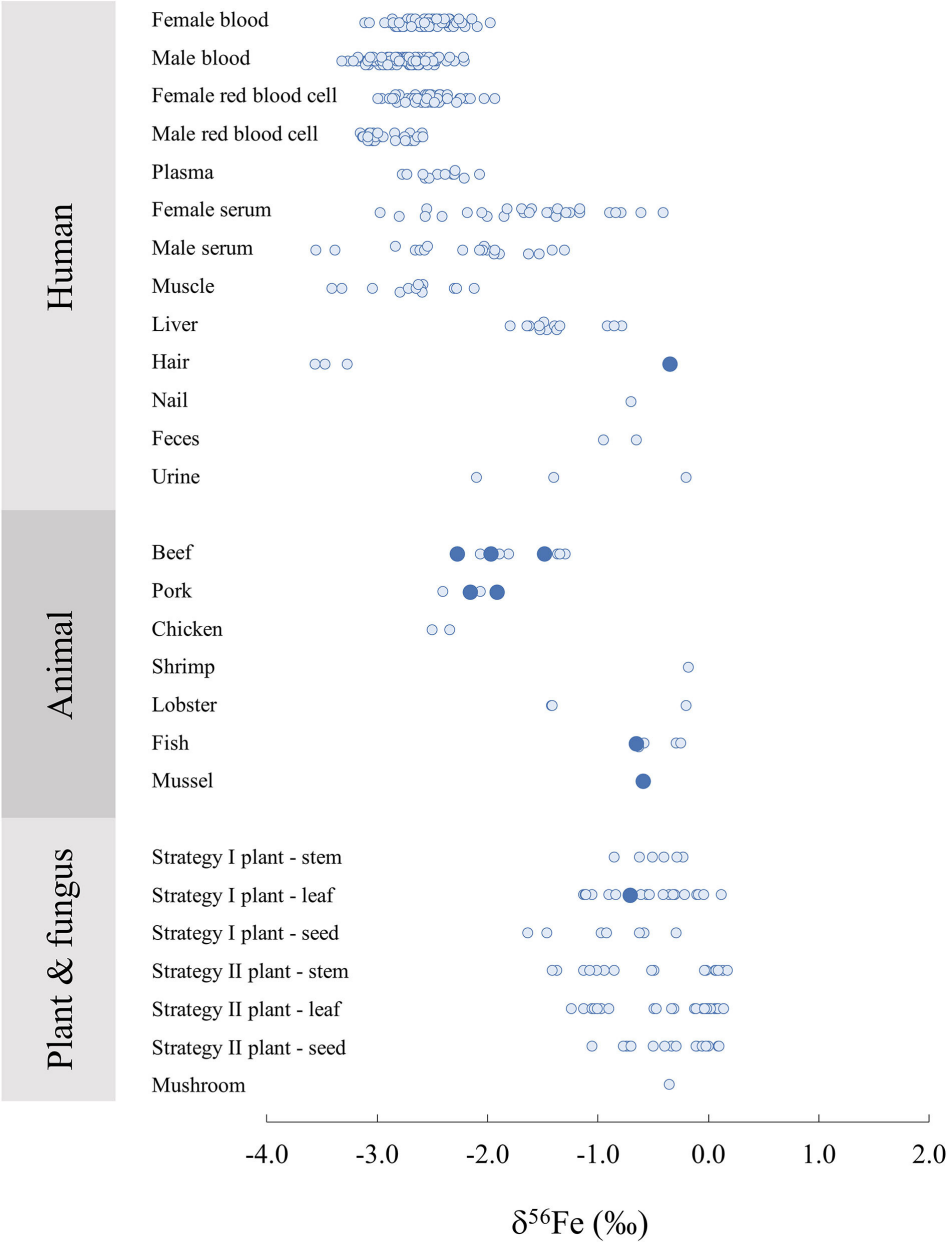
Iron isotope composition δ^56^Fe in biological samples of human, animal, plant and fungus origin from this study (large dark blue circles) and associated literature (20, 27, 34, 36, 44, 48, 51, 52, 56, 59–64).

On Figure 3, δ^56^Fe values are compiled from a large number of studies focusing on biological samples of various origins. Human tissues and organs, although more extensively studied than other biological organisms, lack previous data for Fe isotope composition. In particular, 3 measurements of human hair represented on Figure 3 (36) present very light signatures compared to the reference material measured in this study, suggesting a high variability in Fe isotope compositions in human hair, likely due to environmental variations. Human organ and tissue samples vary significantly amongst individuals as a result of differences in nutritional environments. Moreover, there is a clear difference of Fe isotope compositions between female and male for blood and its components (Figure 3) due to menstruations (43). Male blood and red blood cells present lighter Fe isotope signatures than that of female individuals, which can be explained by an absorption efficiency which is 7% lower in average for male individuals (65). It should be noted that although animal reference materials are widely available, human materials are rarer but would be extremely valuable for cross calibration of isotope data in medical studies.

The two bovine liver reference materials analysed in this study (ERM-BB185) and in (52) (SRM-1577c) have similar δ^56^Fe values within error with −1.48 ± 0.07%0 and −1.33 ± 0.10%0, respectively. Similarly, the bovine muscle reference material measured in this study (ERM-BB184) presents a very similar delta to the beef muscle analysed in Walczyk and von Blanckenburg (36), with −1.97 ± 0.07%0 and −2.06 ± 0.10%0, respectively. On the other hand, the porcine muscle reported in (36) is different from the reference material measured in this study, with −2.4 ± 0.10%0 and −1.92 ± 0.02%0, respectively, suggesting a certain variability. Overall, terrestrial animal organs present consistent δ^56^Fe values reported by several distinct studies, ranging between −2.50%0 and −1.30%0. Marine animal organs also present δ^56^Fe values concentrated in relatively narrow range with globally heavier signatures than terrestrial animals, from −0.63 to −0.18%0, with the exception of a lobster hepatopancreas reference material (TORT-3) with δ^56^Fe = −1.40 ± 0.14%0 (52), which presents a lower δ^56^Fe value than the rest of the marine animal organs.

**Figure 4:**
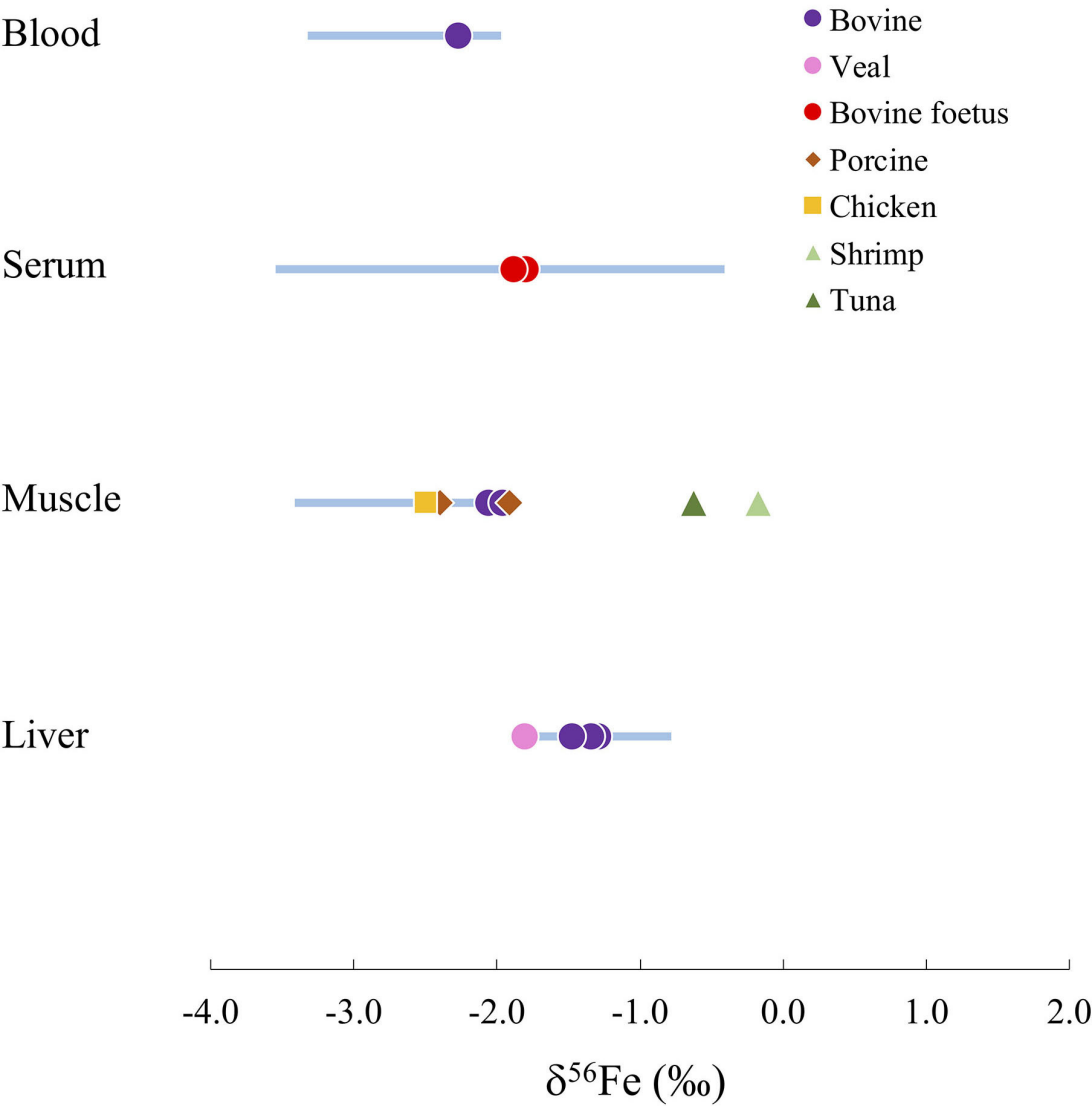
Comparison of the Fe isotope composition δ^56^Fe in different human organs (blue bars) and other animal species (symbols).

For humans and animals, liver samples present heavier Fe isotope signatures than tissues containing Fe^2+^ such as red blood cells and muscle, due to the oxidation of Fe before its storage in ferritin in the liver (59, 60). On Figure 4, the range of Fe isotope compositions of 4 human organs and tissues are compared to the compositions of organs in other terrestrial and marine animal species. Marine animals present isotope compositions persistently heavier than humans. On the other hand, terrestrial animal organs (i.e., beef, pork and chicken) show Fe isotope compositions falling consistently within the range of human organs (Figure 4). This suggests that terrestrial animal organs are well-suited for preliminary investigations of the distribution of Fe isotopes in the body as a result of metabolic pathways with applications to medicine.

Redox reactions involving Fe produce isotope fractionations in plants. In particular, there is a clear difference in Fe isotope composition between strategy I and II, to due a difference in the Fe absorption process from the soil to the plant. Strategy II plants correspond to graminaceous while strategy I plants include all other plant species. When absorbing Fe from the soil, strategy I plants release protons to the soil to reduce the pH to solubilise immobile Fe^3+^ which is reduced to Fe^2+^ in the roots. This process leads to a global light isotope enrichment of the Fe acquired by the plant compared to the soil. On the other hand, graminaceous (Strategy II), release a phytosiderophore complexant that chelate Fe^3+^ which is then absorbed by the root, leading to limited isotopic fractionation between the Fe absorbed by the plant and the soil (20, 34). A clearer difference between organs (stems, leaves and seeds) are also observed for strategy I plants (Figure 3). The δ^56^Fe value of the apple leave standard NIST1515 analysed here (−0.71 ± 0.06%0) falls within the range of the strategy I leaves previously analysed, generally lighter than Strategy II (δ^56^Fe close to zero) and could be used in future plant science studies as data quality control.

## Conclusions

We report novel high-precision Fe isotope MC-ICPMS measurements for nine international biological reference materials: NIST1515 (apple leaves), ERC-CE464 (tuna fish), BCR668 (mussel tissue), ERM-BB184 (bovine muscle), ERM-CE196 (bovine blood), ERMBB-185 (bovine liver), ERM-BB124 (Pork muscle), ERM-BB186 (pig kidney), ERM-DB001 (human hair). For the two standards previously analysed, our data are in excellent agreement with literature. This dataset contains Fe isotope compositions of widely available international reference materials covering a large range of different biological matrices, which will be useful for future works aiming at investigating biological processes through the Fe isotopic system.

## Data Availability Statement

The original contributions presented in the study are included in the article/supplementary material, further inquiries can be directed to the corresponding author/s.

## Author Contributions

The analysis described in this work and associated chemical protocols were performed by first author EK, under the supervision of FM. MP contributed to the digestion of the samples and provided useful comments on the figures. EK wrote the manuscript, with guidance from FM. The fundings of this study were provided by FM and JS. All authors contributed to the article and approved the submitted version.

## Funding

JS thanks the financial support of the French National Research Agency (ANR Project VolTerre, grant no. ANR-14-CE33-0017-01). FM acknowledges funding from the European Research Council under the H2020 framework program/ERC grant agreement 637503 (Pristine), and the ANR through a chaire d'excellence Sorbonne Paris Cité. Parts of this work were supported by IPGP multidisciplinary program PARI, and by Région Île-de-France SESAME Grant no. 12015908, EX047016 and the IdEx Université de Paris grant, ANR-18-IDEX-0001 and the DIM ACAV+.

## Conflict of Interest

The authors declare that the research was conducted in the absence of any commercial or financial relationships that could be construed as a potential conflict of interest.

## Publisher's Note

All claims expressed in this article are solely those of the authors and do not necessarily represent those of their affiliated organizations, or those of the publisher, the editors and the reviewers. Any product that may be evaluated in this article, or claim that may be made by its manufacturer, is not guaranteed or endorsed by the publisher.
